# Spatial distribution and natural environment mechanism of the cholera epidemic in ancient Jiangnan area, China

**DOI:** 10.1371/journal.pone.0248048

**Published:** 2021-03-11

**Authors:** Xiaowei Wang, Mengqi Yang

**Affiliations:** 1 College of Tourism, Shandong Women’s University, Jinan, China; 2 Key Lab of Guangdong for Utilization of Remote Sensing and Geographical Information System, Guangdong Open Laboratory of Geospatial Information Technology and Application, Guangzhou Institute of Geography, Guangzhou, China; 3 Centre for Human Geography and Urban Development, Guangdong Provincial Center for Urban and Migration Studies, School of Geographical Sciences, Guangzhou University, Guangzhou, China; Institute for Advanced Sustainability Studies, GERMANY

## Abstract

Cholera, as an acute, high-risk and widespread infectious disease, has been studied by many scholars. Based on the data from <Annals of Epidemics in China over the Past 3000 Years>, this research investigated the spatial distribution of the cholera epidemic and natural environment mechanism of the cholera epidemic in the Jiangnan area, from the year 1820 to 1821. We applied a set of spatial statistical analyses to investigate the spatial heterogeneity and the factors that influence the cholera epidemic in the Jiangnan area. Results show that: 1) Spatial distribution of cholera epidemic lied at different geographical scales. The cholera epidemic was highly concentrated in Shanghai, Nanjing and Hangzhou; There was a north-south difference of cholera epidemic distribution at the regional scale. The cholera epidemic was more concentrated in the north part than in the south part of the Jiangnan area; Meanwhile, there was an east-west difference in cholera epidemic distribution where the intensity of the cholera epidemic decreased from east to northwest and southwest. 2) A land-sea distribution of cholera can be also found. The Chang-Hang line and the Hu-Jia line were the two boundaries of the cholera epidemic in the Jiangnan area. 3) There was a close relationship between the distribution of the cholera epidemic and natural environment in the Jiangnan area. The influence intensity of natural factors on epidemic disasters followed the order of temperature (0.760) > precipitation (0.663) > river distance (0.413) > river density (0.398) > elevation (0.395). The present investigation is conductive to establish a prevention system for public health emergencies, which contributes to the sustainable development of society and human health.

## 1. Introduction

Cholera is an acute diarrheal infectious disease, induced by Vibrio cholerae [1, 2] pollution of food and water. According to the definition of epidemic disaster [3], a large-scale cholera epidemic is called a cholera disaster. From the historical records, a cholera epidemic broke out in China during the Jiadao period from the year 1820 to 1821 [4, 5], which can be regarded as the first large-scale cholera disaster in China. The cholera epidemic occurred in the Jiangnan area, leading to a highly mortality. The cholera epidemic has been studied extensively from the perspectives of history [6] and sociology [7, 8]. Previous research of the historical cholera epidemic [5] was primarily focused on the epidemic’s origin [4], frequency and significance [9].

Jiangnan area is located in the Yangtze River delta. It is dominated by plains and low hills, and the south part is higher than the north part. Jiangnan area has a subtropical monsoon climate, experiencing humid and rainy weather all the year. Because of the natural environment, the Jiangnan area is densely covered with rivers and lakes, and enjoys a reputation as a “water village”. Flood, drought, and other natural disasters in this area occurred frequently in ancient times [10–12]. Jiangnan area is one of the most populous and important regions in China, with a long history of culture, which has a significant influence on China’s society [13]. In the Jiangnan area, many infectious diseases are environment-related and have existed for hundreds of years, such as malaria [14] and schistosomiasis [15]. The local environment not only affects food security, but also has a great impact on people’s health status [16, 17]. The humid warm weather, densely distributed water systems, and rampant flies in the Jiangnan area are the main causes for the outbreak of the cholera epidemic.

Through combined comprehensive and interdisciplinary analyses, this research investigated the spatial distribution and the mechanism of the cholera epidemic in the Jiangnan area from 1820 to 1821, to establish a prevention system for infectious disease and public health emergency.

## 2. Data and methods

### 2.1 Data sources

#### 2.1.1 Historical data of cholera epidemic

The historical data of the cholera epidemic mainly comes from the <Annals of Epidemics in China over the Past 3000 Years> [18], which is the most detailed collection of epidemic records in China so far. The cholera epidemic during the Jiadao period was a serious epidemic disaster, which became a national health event. Jiangnan area was the origin and the most affected area of this disaster. Extracted the cholera data from historical records in the Jiangnan area from the year 1820 to 1821, this research investigated the relationship between environmental factors and cholera epidemic distribution.

#### 2.1.2 Geographical environment data

Many research [19] has been conducted to stimulate the ancient natural environment based on the modern environment data. This research adopted the same method, in which both the terrain data [20] and the climate data [21] were collected to stimulate the natural environment in ancient times. A spatial analysis tool was used to extract elevation of both administrative divisions and cholera-affected counties from remote sensing image data at the county level. Hydrological data were collected from the <Historical Atlas of China (Volume 8)> [22] for map vectorization.

#### 2.1.3 Administrative division data

Jiangnan area is a geographical area, and a humanistic social area as well. The boundary for the Jiangnan area changes continuously [23]. In this research, the Jiangnan area included 9 prefectures (city-level administration): Suzhou, Songjiang (Shanghai), Changzhou, Zhenjiang, Jiangning (Nanjing), Hangzhou, Jiaxing, Huzhou, and Taicang [24]. The year 1820 was chosen as the standard year. The data from the <Historical Atlas of China (Volume 8)> [22] and the <Comprehensive Table of the Evolution of the Qing Dynasty’s Political Districts> [25] were collected to draw a geographical base map to illustrate the administrative divisions of ancient Jiangnan area.

### 2.2 Research methods

The cholera epidemic data of this research were collected from historical records. Because the raw data were qualitatively described, it is difficult to run quantitative statistical analyses. This research constructed a cholera epidemic database, converted the qualitative description into quantitative data.

The adopted research model and geographical interpretation were summarized in Table 1. To understand the relationship between the cholera epidemic and its spatial distribution, this paper employed the center of gravity method, the epidemic intensity, the nearest neighbor index, the geographic concentration index, and the imbalance index. The spatial characteristics of the cholera epidemic (i.e., spatial distribution, spatial distribution equilibrium, and spatial distribution correlation) can be assessed. This research also applied the Geographic Information System (GIS) spatial coupling analysis tools and geographic detector to detect the environmental influencing factors and to analyze their impact on the cholera epidemic.

**Table 1:** Research model and interpretation of geographical significance.

Mathematical model	Model interpretation	Geographical significance
Nearest neighbor index: $R=\frac{2\sum_{i=1}^{n}d_{i}\sqrt{\frac{n}{A}}}{n}$ (1)	*d*_*i*_ equals to the distance between element *i* and its nearest neighbor, *n* equals to the total number of points, and *A* is the area.	The nearest neighbor index method is a basic method to understand the distribution of geographical subjects [26]. There are three types of spatial distribution of point elements: uniform, concentrated and random [27]. When *R* = 1, it tends to be randomly distributed; when *R*>1, it tends to be evenly distributed; when *R*<1, it tends to be aggregated.
Geographic concentration index: $G=100*\sqrt{\sum_{i=1}^{n}{\left(\frac{X_{i}}{T}\right)}^{2}}$ (2)	*n* is the total number of regions, *T* is the total number of elements *i*, and *X*_*i*_ is the number of elements *i* in the region.	The geographic concentration index is an important indicator that reflects the concentration of geographic subjects [28]. *G*∈(0–100), the larger the *G* value, the more concentrated the distribution of elements; the smaller the *G* value, the more dispersed the distribution.
Imbalance index: $S=\frac{100\sum_{i=1}^{n}Y_{i}-50\left(n+1\right)}{100n-50\left(n+1\right)}$ (3)	*n* is the total number of regions, and *Y*_*i*_ is the cumulative percentage of the place. After all elements in the region are sorted following a descending order. It reflects the balanced degree of the distribution of elements in the region.	The imbalance index reflects the degree of equilibrium of the distribution of research objects in different regions [29]. If *S* = 0, it is evenly distributed in each city; if *S* = 1, it is all concentrated in one city, and the spatial distribution is extremely unbalanced, where *S* ∈(0–1).
Center of gravity: ${\bar{X}}_{w}=\frac{\sum_{i=1}^{n}w_{i}x_{i}}{\sum_{i=1}^{n}w_{i}},{\bar{Y}}_{w}=\frac{\sum_{i=1}^{n}w_{i}y_{i}}{\sum_{i=1}^{n}w_{i}}$ (4)	*x*_*i*_ and *y*_*i*_ are the coordinates of element *i*, *n* is the total number of element *i*, and *w*_*i*_ is the weight of element *i*.	The geographic center of gravity method can identify the geographic center (or density center) of a group of geographic subjects, and can be used to analyze the distribution and change of geographic subjects.
Epidemic intensity: I=0.25F'+0.25B'+0.25D'+0.25T' (5)	*F* is the frequency of the epidemic, *B* is the breadth of the epidemic, *D* is the density of the epidemic, *T* is the thickness of the epidemic [30–34], and *F*’, *B*’, *D*’ and *T*’ are their standardized values.	The intensity of the epidemic reflects the severity of the epidemic in a specific area during a specific period [35].
Differentiation and factor detection: $\begin{array}{l} q=1-\frac{\sum_{h=1}^{L}N_{h}\sigma_{h}^{2}}{N\sigma^{2}}=1-\frac{SSW}{SST}\\ SSW=\sum_{h=1}^{L}N_{h}\sigma_{h}^{2},SST=N\sigma^{2} \end{array}$ (6)	*h* = 1,. . ., *L* is the stratification of variable Y or factor X, that is, classification or partition; *N*_*h*_ and *N* are the number of units in layer *h* and the whole area, respectively; $\sigma_{h}^{2}$ and *σ*^2^ are the layers *h* and Y in the whole area, respectively; The variances *SSW* and *SST* are the sum of variance within the layer and the total variance of the whole region, respectively.	The value of *q* is [0, 1], and the larger the value, the more obvious the spatial differentiation of Y; if the stratification is generated by the independent variable X, the larger the value of *q*, the stronger the explanatory power of the independent variable X on the attribute Y; otherwise it is weak. If *q* = 1, factor X completely controls the spatial distribution of Y, whereas *q* = 0 means that factor X has no relationship with Y [36].

## 3. Results

### 3.1 Characteristics of spatial distribution of the cholera epidemic in the Jiangnan area

In this section, the results of spatial distribution, spatial distribution equilibrium and spatial distribution correlation are presented.

#### 3.1.1 Spatial distribution

The nearest neighbor index method measures the distance between geographical subjects (formula (1)). Results indicated that the average observation distance of 11.27 km was less than the theoretical average distance of 17.45 km, and the nearest neighbor index was determined as R = 0.646<1. Each cholera-affected county was marked as a spot. Cholera-affected counties were clustered into various groups of spot. The spot distances within the same group were relatively small, while it was far from each other between different groups. For example, Suzhou and Songjiang prefecture both had the cholera epidemic, and cholera-affected counties in these two prefectures were closely connected.

#### 3.1.2 Spatial distribution equilibrium

The geographic concentration index (formula (2)) of cholera-affected counties in the Jiangnan area was calculated (G = 37.12), which was higher than the average geographic concentration index (G = 33.33). It indicated that cholera-affected counties were concentrated during the Jiadao period at the county level. The imbalance index of cholera-affected counties (S = 0.30>0) was calculated based on (formula (3)). The result showed that cholera-affected counties were unevenly distributed. Suzhou, Songjiang, and Changzhou prefecture accounted for 54.35% of the total number of cholera-affected counties in the Jiangnan area; Jiangning, Zhenjiang, and Hangzhou prefecture accounted for 17.39% of the total number of cholera-affected counties in the Jiangnan area.

Analyses of both concentration index and imbalance index indicate an uneven distribution of cholera epidemic in Jiangnan area.

#### 3.1.3 Spatial distribution correlation

**Fig 1:**
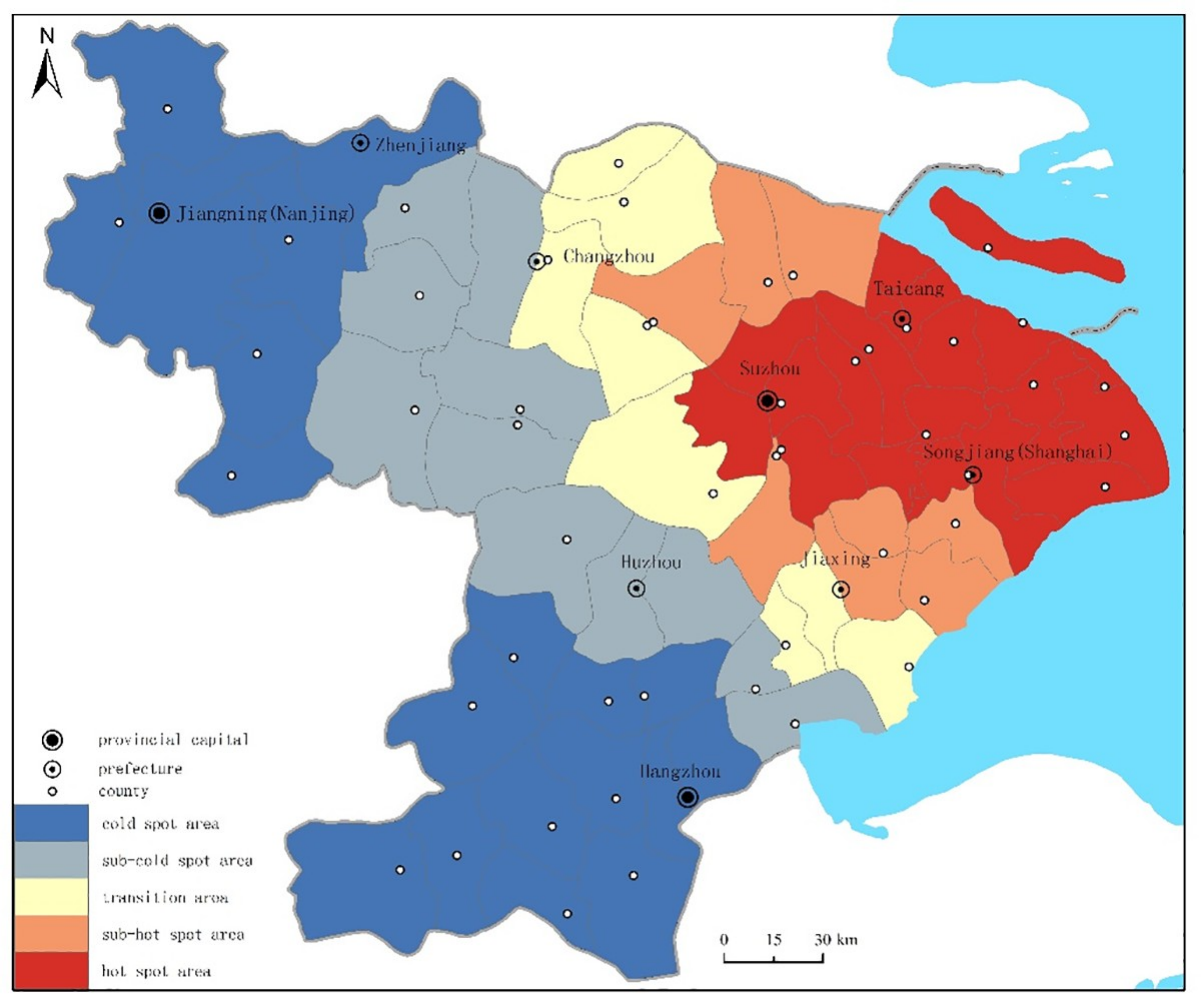
Hot and cold spots of the cholera epidemic in the Jiangnan area from 1820 to 1821 (Copyright hold by the authors).

This research applied the geographic detector Getis-Ord Gi* to measure the spatial correlation of the cholera epidemic in the Jiangnan area. The natural breakpoint classification method (Jenks) was used to divide the Jiangnan area into 5 classes, and the values were ranked in descending order as shown in Fig 1 as follows: hot spot area, sub-hot spot area, transition zone, sub-cold spot area, cold spot area. There was an east-west difference in cholera epidemic distribution in the Jiangnan area. Results showed that statistically significant high-high (hot spot area) areas were clustered in the east of Jiangnan area, while the low-low (cold spot area) areas were clustered in the west of the Jiangnan area. From east to west, different hot zones can be identified as follows: hot spot area, sub-hot spot area, transition zone, sub-cold spot area and cold spot area.

The high clustered areas and the low clustered areas were compared as tabulated in Table 2. It was found that 24 counties for hot spot / sub-hot spot areas accounted for 53.33% of the total number of cholera-affected counties in the Jiangnan area, among which, 16 counties belonged to hot spot areas and accounted for 35.56% of the total number of cholera-affected counties. On the contrary, 15 counties for cold spot / sub-cold spot areas accounted for 33.33% of the total number of cholera-affected counties in the Jiangnan area, among which, 7 counties belonged to cold spot areas and accounted for 15.56% of the total number of cholera-affected counties in the Jiangnan area. A core-edge structure was centered in Songjiang (Shanghai), Hangzhou, and Jiangning (Nanjing) prefecture, among which Songjiang prefecture had the highest value among hot spot areas. Hangzhou and Jiangning prefecture had the lowest value among cold spot areas. The geographical distribution of hot-cold spot area indicated an east-west distribution of cholera epidemic in the Jiangnan area.

**Table 2:** Distribution of hot and cold spots of the cholera epidemic at the county level in the Jiangnan area from 1820 to 1821.

Hot/cold spot area	County number	Percentage (%)
Cold spot area	7	15.56
Sub-cold spot area	8	17.78
Transition area	6	13.33
Sub-hot spot area	8	17.78
Hot spot area	16	35.56

### 3.2 Spatial distribution of the cholera epidemic in the Jiangnan area

#### 3.2.1 North-south distribution of the cholera epidemic

In this section, spatial heterogeneity is mainly used to study the spatial distribution of cholera disease. One of the major research questions is to understand the geographical distribution of the cholera epidemic in the Jiangnan area. Taking both administrative division and population distribution into consideration, this research is the very first to draw a geographical boundary of cholera in the Jiangnan area.

At the regional scale, there was a north-south difference for the distribution of the cholera epidemic. Jiangnan area consisted of the southern Jiangsu area and the northern Zhejiang area. Southern Jiangsu area included Suzhou, Songjiang, Changzhou, Taicang, Jiangning, and Zhenjiang prefecture; Northern Zhejiang area included Hangzhou, Jiaxing, and Huzhou prefecture. Results showed that there were more epidemic disasters in the northern Jiangnan area than in the southern Jiangnan area. There were 32 cholera-affected counties in the southern Jiangsu area, and the number of cholera-affected counties accounted for 11.85% of the total number of cholera-affected counties in the Jiangnan area. There were13 cholera-affected counties located in the northern Zhejiang area, which accounted for 9.63% of the total number of cholera-affected counties in the Jiangnan area. Results indicated that there were more cholera diseases in the north and fewer cholera diseases in the south.

**Fig 2:**
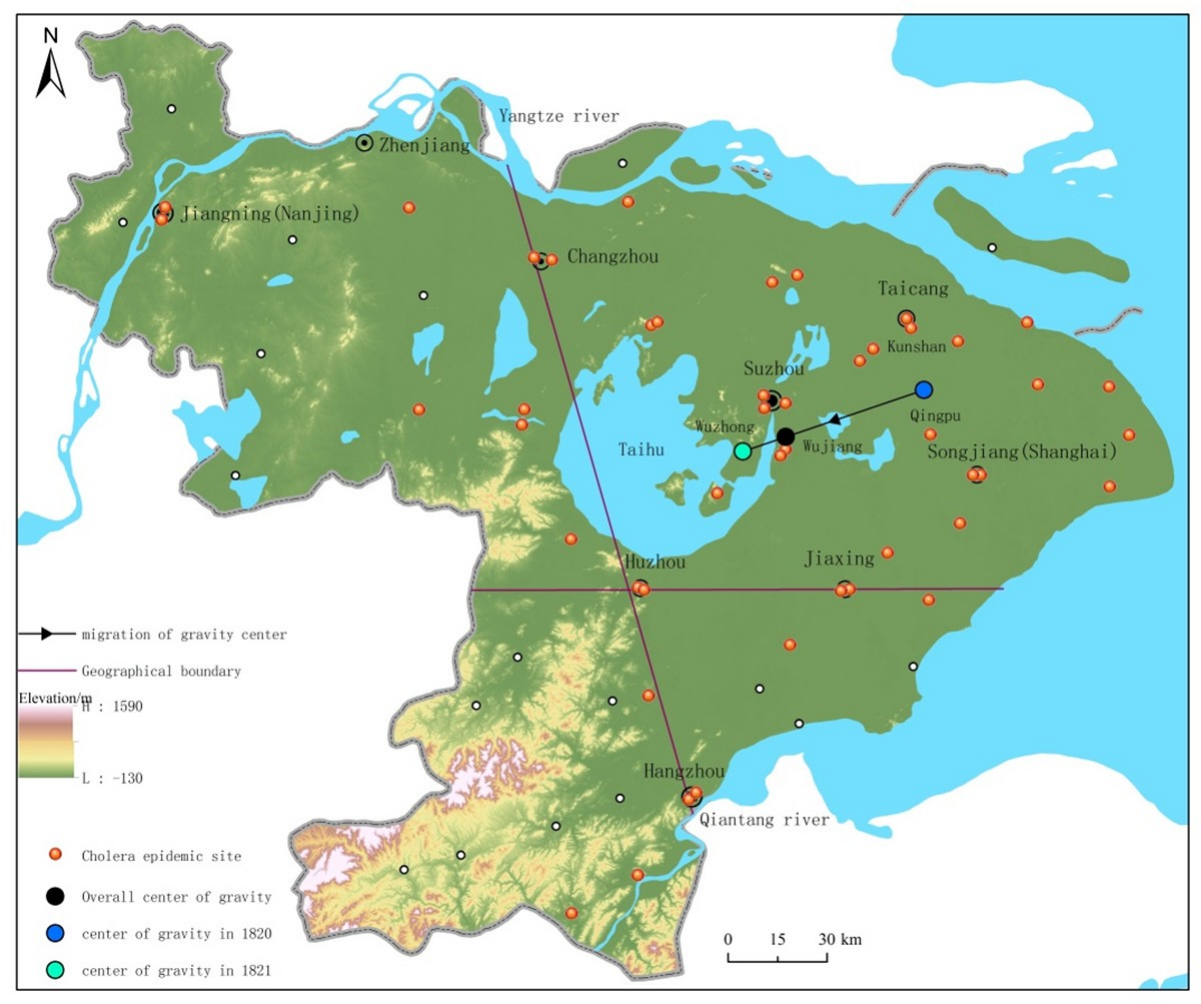
Geographical distribution of the cholera epidemic in the Jiangnan area from 1820 to 1821 (Copyright hold by the authors).

The center of gravity (formula (4)) calculated the coordinates of the geographical center of cholera-affected counties. All derived coordinates were located on the geographical base map of the Jiangnan area (Fig 2). Results showed, in 1820, the gravity center of the cholera epidemic was between Qingpu and Kunshan county (121°7’6.955” E, 31° 16’35.873” N). In 1821, the gravity center of cholera epidemic moved westward to Wuzhong county (120°29’45.09"E, 31°10’50.621"N). The overall gravity center between Wujiang and Wuzhong county (120°39′11.279″E, 31°12′28.258″). All gravity centers were located in the southern Jiangsu area. Results showed that the cholera epidemic mainly occurred in southern Jiangsu area.

#### 3.2.2 Distribution characteristics of the cholera epidemic

**Fig 3:**
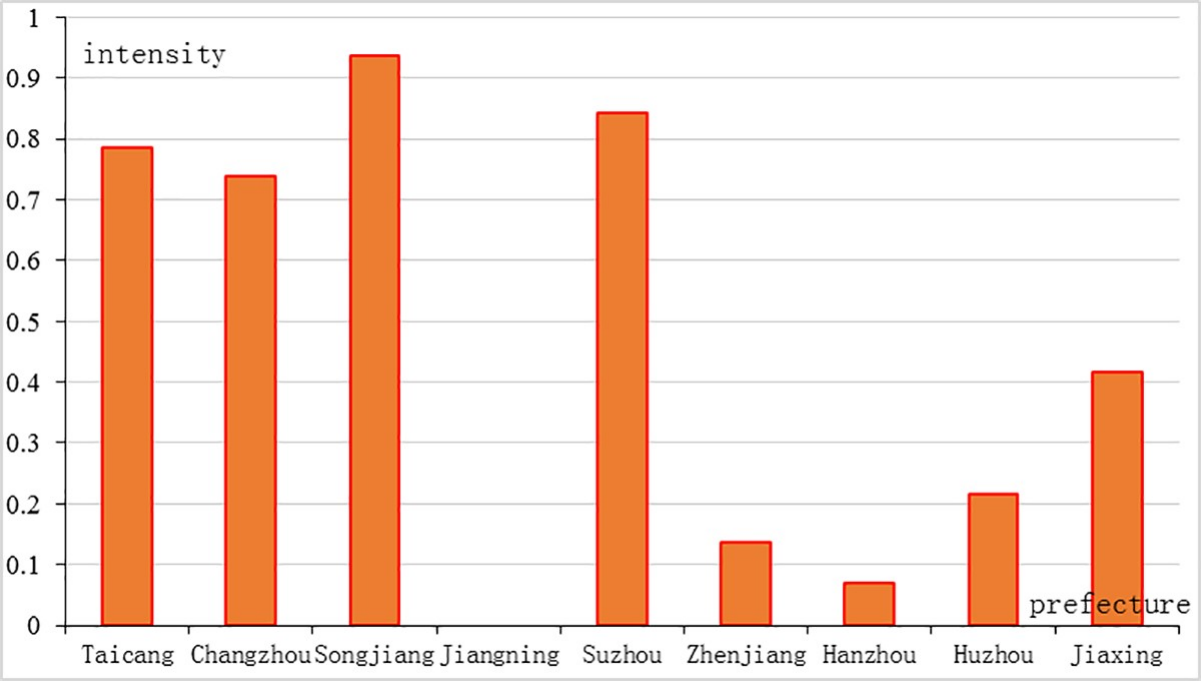
Epidemic intensity of the cholera epidemic in the Jiangnan area from 1820 to 1821.

The intensity of the cholera epidemic reflects the severity of epidemic in a specific area during a certain period. At the prefectural scale, the epidemic intensity was calculated (Fig 3) (formula (5)). The cholera epidemic distribution in the southern Jiangnan area shows a decreasing trend from east to the northwest and southwest. As shown in Fig 3, Songjiang prefecture had the highest epidemic intensity (0.94), while Hangzhou (0.071) and Jiangning (Nanjing, 0.00) prefecture had two of the lowest epidemic intensities.

The origin of the cholera epidemic can be speculated based on the interpretation of the decreasing trend. During the Kangxi period (1662–1722), Shanghai county was first established as a local port. During the Qianlong and Jiaqing period (1736–1820), it gradually became a national commercial port and a grain logistical center. During the Jiadao period (1820–1821), not only famous for its business reputation, but Shanghai county was also known as the origin of the cholera epidemic. Result indicated that Songjiang prefecture, where Shanghai county was located, had the highest cholera epidemic intensity in the Jiangnan area. And Shanghai county had the highest intensity in the Songjiang prefecture.

The severity of cholera decreased with distances from Shanghai county. Results indicated that the distance from epidemic origin was more significant on the distribution of cholera epidemic than population. In 1820, the population of the northwest Jiangning prefecture was 5.252 million, and the population of the southwest Hangzhou prefecture was 3.197 million [36]. These two areas had similar population with Shanghai county. Because the distance of the two areas were far from the epidemic origin, the epidemic intensities were much lower than Shanghai county.

#### 3.2.3 Land-sea distribution of cholera epidemic

The estuaries of Yangtze River and Qiantang River encircled a coastal hinterland. Changzhou county and Hangzhou county were the two counties that located near the estuaries. We drew a line between Changzhou county and Hangzhou county and named it Chang-Hang line (Fig 2). Results showed there was a significant land-sea difference in the cholera epidemic distribution in the Jiangnan area. There were 35 cholera-affected counties located on the east side of the Chang-Hang line, which accounted for 77.78% of the total number of cholera-affected counties. This area was located in the impact plain of the Yangtze River delta. The Yangtze River and the Qiantang River flow into the East China Sea. This terrain was featured by low land and intensive water networks. The combination of alkaline river-water, suitable temperature, and humidity was conducive to the survival and reproduction of Vibrio cholerae. Similarly, Huzhou county and Jiaxing county were the two counties that located at the division between the southern Jiangsu area and the northern Zhejiang area. We drew a line between Huzhou county and Jiaxing county and named it Hu-Jia line (Fig 2). A fan-shaped area (Fig 2) was encircled by the Chang-Hang line, the Hu-Jia line and the coastline. There were 27 cholera-affected counties in this fan-shaped area, accounted for 60% of the total number of cholera-affected counties. In terms of epidemic intensity, it was the most cholera-affected area, where Songjiang prefecture (0.94), Suzhou prefecture (0.84), Taicang prefecture (0.78), and Changzhou prefecture (0.74) ranked the top four cholera-affected prefectures. From the results, the Chang-Hang line and the Hu-Jia line were geographical boundaries of the cholera epidemic in the Jiangnan area.

### 3.3 Natural environment mechanism of the cholera epidemic

Modern science has proved that many factors can affect the cholera epidemic. Climatic and geographical factors play significant roles in determining the cholera epidemic [37]. Topographic factors, climatic factors, and hydrological factors were therefore selected to investigate the influence on cholera distribution in this research.

#### 3.3.1 Terrain factor

In this research, the topographical data were divided into 5 classes. The elevation topographic map (DEM) was overlaid on the cholera epidemic distribution map of the Jiangnan area to investigate whether there were any relationship between the cholera epidemic and elevation.

Results showed that the cholera epidemic was more likely to occur in lowland. It was because early settlements for human beings were concentrated in plains [38]. Table 2 shows that there were 5 cholera-affected counties with an elevation below 5 m, accounted for 11.11% of the total number of the cholera-affected counties; There were 32 cholera-affected counties with an elevation below 10 m, accounted for 71.11% of the total number of the cholera-affected counties; and there were 41 cholera-affected counties with an elevation below 20 m, accounted for 91.11% of the total number of cholera-affected counties. On the other hand, there were 4 cholera-affected counties with an elevation above 20 m, accounted for 8.89% of the total number of cholera-affected counties.

Results also indicated that geographical boundaries, mountains, prevented cholera epidemic from pervasion. There was no evidence of the cholera epidemic in mountainous areas. No cholera record was found in Yuqian county, Changhua county, Lin’an county, or Xiaofeng county where they were the mountainous area in the Jiangnan area.

#### 3.3.2 Climate factors

Jiangnan area located in the subtropical zone. The suitable temperature and precipitation provided a comfortable environment for the survival and reproduction of Vibrio cholerae [39]. The interpolation method was used to extract average annual temperature and precipitation of places with the cholera epidemic.

In terms of temperature, the cholera epidemic was more likely to occur in cold places rather than in hot places. The Vibrio cholerae was sensitive to heat and dryness [40]. Results showed that 88.89% of the cholera-affected counties with an average annual temperature below 16.92°C.; There were 27 cholera-affected counties with an average annual temperature below 16.74, accounted for 60% of the total number of cholera-affected counties, And there were 16 cholera-affected counties with an average annual temperature below 16.52, accounted for 35.56% of the total number of the cholera-affected counties in the Jiangnan area.

In terms of precipitation, the cholera epidemic was more likely to occur in humid places rather than in dry places. Results showed that there were 35 epidemic-affected counties with an average annual rainfall above1130 mm and accounted for 77.77% of the total number of the cholera-affected counties; The cholera-affected counties with an average annual rainfall below 1080 mm only accounted for 4.44% of the total number of the cholera-affected counties in the Jiangnan area.

Results indicated that the Vibrio cholerae had a strong dependency on water. The tropical cyclones moved to the north-west Pacific Ocean and landed in the Jiangnan area, with storm surges. The Meiyu front-heavy rain process brought a large amount of precipitation and caused flood. The large amount of river water was polluted with Vibrio cholerae [10]. People drank polluted river water and got infected. According to the historical record, in the twenty-fifth year of Jiaqing period (1820), there was serious blood on July 27, people drank the polluted river water and got sick. A cholera epidemic then broke out in Hepu county in the following August.

#### 3.3.3 Hydrological factors

River systems play a significant role in the distribution of the cholera epidemic. Results shown in Table 3, with an increasing of river density, it was more like to have cholera-affected counties in the Jiangnan area. Within 3 km of a river, there were 35 cholera-affected counties, accounted for 77.78% of the total number of cholera-affected counties; The 51.11% of the cholera-affected counties were within 2 km of a river in the Jiangnan area.

**Table 3:** Spatial coupling relationship between epidemic county quantity and natural environment division.

Variable	Elevation (m)				
	<5	6–10	11–20	21–30	>31
Percentage of affected counties (%)	11.11	60.00	20.00	6.67	2.22
Variable	Temperature (°C)				
	<16.52	16.52–16.74	16.74–16.96	16.96–17.18	>17.18
Percentage of affected counties (%)	35.56	24.44	32.50	2.22	8.89
Variable	Precipitation (mm)				
	<1080	1080–1130	1130–1180	1180–1230	>1230
Percentage of affected counties (%)	4.44	17.78	33.33	13.33	31.11
Variable	River distance (km)				
	<1.00	1–2.00	2–3	3–5	>5
Percentage of affected counties (%)	28.89	22.22	26.67	6.67	15.56
Variable	River network density (m/km^2^)				
	<30	30–55	55–75	75–100	>100
Percentage of affected counties (%)	4.44	17.78	17.78	11.11	48.89

This research measured both the shortest distance from the water source to the cholera-affected county and the total length of the water network in the county, and calculated the density of the river network. In terms of the density of the river network, there were 35 cholera-affected counties had a density of river network above 55 m/km^2^, accounted for 77.78% of the total number of cholera-affected counties; The 48.89% of the cholera-affected counties had a density of river network above 100 m/km^2^ in the Jiangnan area.

The Jiangnan area is covered with densely distributed water systems, such as the Yangtze River, Qiantang River, and Taihu Lake. Due to the lack of water purification system, people had to drain waste-water into river without purification. Toilet flushing water directly polluted into river. The Vibrio cholerae polluted river water caused bluebottle to breed. From this, people’s daily life-style made the situation worse, and aggravated the cholera epidemic [41]. Besides, small boats were the main vehicles for daily transportation in the Jiangnan area in ancient times. It is likely to transmit Vibrio cholerae though water channels by boats.

#### 3.3.4 Impact evaluation

The Geodetector was used to detect the influencing factors of the cholera-affected areas in this research. Because of the advantage for processing variables, the Geodetector is widely used in public health [42–44] and urban-rural geography research [44, 45].

This research quantified the effect of influencing factors of cholera by comparing the accumulated dispersion variance of each stratum with the dispersion variance of the Jiangnan area. The F-test method was used to demonstrate whether the accumulated variance of each stratum was significantly different from the variance in the Jiangnan area. Table 3 presented the power of each influencing factors of the cholera disease. Q-statistics in Geodetector is an indicator for the contribution to the spatial heterogeneity of the cholera disease, where a higher q value indicates a stronger contribution. The factor detector formula (6) can detect the explanatory power of factors on geographical subjects. Results (Table 4) showed that natural factors influenced the spatial distribution of the cholera epidemic in the Jiangnan area. The influencing intensities of natural factors of the cholera epidemic followed the order of temperature (0.760), precipitation (0.663), river distance (0.413), river density (0.398), and elevation (0.395). Climate factors had the greatest intensity, followed by hydrological factors and topographic factors. Results suggested that all factors influenced the distribution of cholera disease. Climate factor (temperature) had the greatest influence on the intensity of cholera epidemic, followed by hydrological factors (precipitation, river distance, river density) and topographic factor (elevation).

**Table 4:** Q-statistics of influencing factors.

Influencing factors	q-statistics
Temperature	0.760
Precipitation	0.663
River distance	0.413
River density	0.398
Elevation	0.395

The interaction between different influencing factors was presented in Table 5. The q-statistics values of X1, X2, X3, X4, and X5 represented the precipitation, temperature, elevation, river density, and river distance respectively. Results showed that all interactive q-statistics values were greater than the value of a single factor. For instance, the q-statistics value of X1 was 0.66, which was smaller than any combinations between X1 and other influencing factors. This demonstrated that the interplay influence of factors played a significant role in the spatial distribution of the cholera epidemic in the Jiangnan area.

**Table 5:** Interaction between influencing factors.

	X1	X2	X3	X4	X5	X6
X1	0.66					
X2	0.95	0.76				
X3	0.99	0.93	0.40			
X4	0.93	0.99	0.99	0.40		
X5	0.99	0.99	0.97	0.99	0.41	

X1: Precipitation, X2: Temperature, X3: Elevation, X4: River density, X5: River distance.

## 4. Discussion

In this research, the most accurate and authoritative historical materials of the cholera epidemic were collected. Because of data-integrity and consistency, the uncertainty in data is inevitable. Having modern terrain data and climate data, a picture of the natural environment of the ancient Jiangnan area is taken, which helps to investigate the association between the spatial distribution of cholera epidemic and the natural environment. The use of the modern method to simulate the historical natural environment is useful, however, there are certain limitations for the mathematical methods. Further statistical research is needed to be done.

Many factors contribute to the outbreak of the cholera epidemic, and influence the distribution of the cholera epidemic. In this research, the physical geography factors are taken into consideration. The health influence of population’s demographic characteristics, social-economic status and interaction of health related factors on the cholera epidemic should be investigated in the future.

## 5. Conclusions

In the present work, the spatial distribution and the natural environmental mechanism of the cholera epidemic in the Jiangnan area from 1820 to 1821 are systematically investigated. The following conclusions can be drawn:

1. The distribution of the cholera epidemic was geographically uneven. About 54.35% of the total cholera-affected counties in the Jiangnan area were concentrated in Suzhou, Songjiang, and Changzhou prefectures. A north-south difference in cholera epidemic distribution in the Jiangnan area was significantly different among regions.
2. There was a land-sea difference of distribution for the cholera epidemic. The cholera epidemic was likely to break out near water areas. The Chang-Hang line and the Hu-Jia line were the geographical boundaries of the cholera epidemic in the Jiangnan area.
3. The physical geography factors had a significant influence on the cholera epidemic in the Jiangnan area. The humid and low land areas were most likely to have cholera epidemic. The intensity of influencing factors were: temperature (0.760)> precipitation (0.663)> river distance (0.413)> river density (0.398)> elevation (0.395).
4. During the Jiaodao period (1820–1821), cholera epidemic was widespread in China. Suzhou and Songjiang prefecture experienced the most serious cholera epidemic in the Jiangnan area. Big cities played the most significant role in preventing epidemic. Establishing a health protection system and improving public facilities are crucial to prevent public health events.

## References

[1] XIAO Wen-jia, WU Huan-yu, GONG Xiao-huan, et al. Epidemiological characteristics of cholera in Shanghai city, 2008–2017. Chin J Public Health, 2019, 35(6): 750–754.

[2] SUN Qi, Meng ZHANG, QI Feng-qing, et al. Research status of cholera based on the data from SCI databases. Modern Preventive Medicine, 2016, 43(21): 3993–4002.

[3] Sheng-sheng GONG. Changes of the temporal-spatial distribution of epidemic disasters in 770BC-AD1911 China. Acta Geographica Sinica, 2003, 58(6): 870–878.

[4] Li SHAN. The analysis about the original problem of cholera in China. Journal of Chinese Historical Geography. 2014, 29(1): 48–56.

[5] WANG Xiao-wei, LI Zi-mo. Bibliometric Analysis of the Progress in Epidemic Research in China. Tropical Geography, 2015, 35(2): 186–192.

[6] Bing-tao LIU. The cholera of Shaanxi province and social response in 1932. Journal of Chinese Historical Geography, 2010, 25(3): 113–124.

[7] Ping ZHANG. Epidemic disease research from the perspective of environment history: Three Problems Resulted from Cholera in Shanxi in 1932. Qinghai Journal of Ethnology, 2014, 25(3): 154–160.

[8] Ping ZHANG. The spread of plague and environmental disturbance in a vulnerable environment: A case study of the Shanxi cholera outbreak of 1932. Historical Research, 2017(2):73–87.

[9] Li SHAN. Frequency of cholera pandemic in China based on local gazetteer: Discussion around the significance of modern history about the first cholera pandemic. Journal of Chinese Historical Geography, 2017, 32(1): 146–158.

[10] GE Yun-jian, WU Xiao-han. Spatial and temporal distribution of historical flood disasters in Jiangsu province. Resources and Environment in the Yangtze Basin, 2019, 28(8): 1998–2007.

[11] Shan-feng HE, Quan-sheng GE, Shaohong WU, et al. Projecting Spatio-temporal Patterns of Flood Hazard over Anhui Province. Chinese Journal of Population, Resources and Environment, 2012, 22(11): 32–39.

[12] HE Shan-feng GE Quan-sheng, Wu Shao-hong, et al. Projecting Spatio-temporal Patterns of Drought Hazard in Southwest China under SRES B2 Scenario. Chinese Journal of Population, Resources and Environment, 2013, 23(9): 165–171.

[13] GE Da-zhuan, Hua-lou LONG, Ying-nan ZHANG, et al. Pattern and coupling relationship between grain yield and agricultural labor changes at county level in China. Acta Geographica Sinica, 2017,72(06):1063–1077.

[14] ZHANG Jing, WANG Cheng-xin, BAI Hu-qun, et al. Trend and influence factors of zoonosis-borne diseases in the population of three gorges area. Resources and Environment in the Yangtze Basin, 2004, 13(2): 145–148.

[15] ZHAO An, LIU Qing, ZHANG Gang-gang, et al. Multiple correlation analysis between schistosomiasis epidemic and environmental factors on the basis of empirical study in the administrative villages of nanchang county. Resources and Environment in the Yangtze Basin, 2015, 24(2): 257–262.

[16] Xin-zhong YU. Epidemics in Jiangnan during the Jiaqing and Daoguang regions. Studies in Qing History, 2001(2): 1–18. 19697487

[17] Yang M, Zhang C. Comparative study on retrofitting strategies for residential buildings after earthquakes[J]. Earthquakes and Structures, 2019, 16(4) 375–389.

[18] Sheng-sheng GONG. Annals of Epidemics in China over the Past 3000 Years (in Chinese). Jinan: QiLu Press, 2019. ISBN 978-7-5333-3795-7.

[19] Long CHENG. Grain raising and border defense in the Northern Song Dynasty——Take the North China war zone as an example. Beijing: commercial press, 2012: 89.

[20] Resource and environment science and data center of Chinese Academy of Sciences, http://www.resdc.cn/data.aspx?DATAID = 217.

[21] National Meteorological information center, http://data.cma.cn/data/cdcdetail/dataCode/A.0029.0005.html.

[22] Qi-xiang TAN. The historical atlas of China. Beijing: SinoMaps Press, 1996.

[23] Zhen-he ZHOU. Interpretation of Jiangnan. Journal of Chinese Literature and History, 1992(49): 141–147.

[24] Bo-zhong LI. On the definition of "Jiangnan region". Journal of Chinese Social and Economic History, 1991(1): 100–105.

[25] Ping-han NIU. A comprehensive table of the evolution of the political districts in the Qing Dynasty. Beijing: SinoMaps Press, 1997: 120–121, 123–124, 138.

[26] TOBLER W R. A computer movie simulating urban growth in the Detroit region. Economic Geography, 1970, 46(2): 234–240.

[27] Ping LIU, Ji-ping LIU, ZHAO Rong, et al. Determination of Lancang River Basin’ s residential distribution type by ddjacent-point index method. Bulletin of Surveying and Mapping, 2007 (10): 36–39.

[28] Ji-gang BAO, ZHENG Hai-yan, Guang-quan DAI. The evolvement of spatial structure and the significance of Guilin’s domestic tourist origins. Acta Geographica Sinica, 2002, 57(1): 96–106.

[29] Li ZHOU, Dian-ting WU, Ze-hong LI, et al. Study on spatial distribution and its influencing factors of natural tourism resource in China-Mongolia-Russia Economic Corridor. Resources Science, 2018, 40(11): 2168–2176.

[30] WANG Xiao-wei, GONG Sheng-sheng, Zi-mo LI, et al. Relationship between the spatial pattern of epidemic disasters and environment in China from 960 to 1368. Journal of Arid Land Resources and Environment, 2021, 35(1): 70–75.

[31] GONG Sheng-sheng WANG Xiao-wei, GONG Chongya. A geographic study of epidemic disasters in China from 1279 to 1368. Journal of Chinese Historical Geography, 2015, 30(2): 24–40.

[32] Sheng-sheng GONG, Xiao-wei WANG, Tao ZHANG. A geographic study of epidemic disasters of Jiangnan area in the Ming Dynasty(1368–1644). Geographical research, 2014, 33(8): 1569–1578.

[33] Sheng-sheng GONG, Zi-mo LI, Guo-xu LIU, et al. Temporal-spatial evolution of war-epidemic disasters in historical China. Journal of Catastrophology, 2019, 34(1): 78–86.

[34] Xiao-wei WANG, Sheng-sheng GONG. Studies on epidemic disasters of Jiangnan area in the Qing Dynasty(1644–1911). Journal of Chinese Historical Geography, 2015, 30(3): 18–30.

[35] Sheng-sheng GONG, Zi-mo LI. Temporal-spatial distribution of epidemic disasters of Shanxi in the Qing Dynasty. Journal of Arid Land Resources and Environment, 2017, 31(6): 1–6.

[36] WANG Jin-feng, Cheng-dong XU. Geodetector: Principle and prospective. Acta Geographica Sinica, 2017, 72(1): 116–134.

[37] Wan-sheng GU. Introduction to Preventive Medicine. Shanghai: Shanghai Science and Technology Press, 1988: 105

[38] WANG Xiao-wei HE Xiao-qian, GE Da-zhuan, et al. Review on Literature of Historical Settlement Geography in China. Tropical Geography, 2012, 32(1): 107–112.

[39] WANG Tao SHEN Wei-shou, LIN Nai-feng, et al. Changes in ≥0°C Accumulated Temperature and Agricultural Adaptative Strategies in the Yangtze River Basin in Recent 50 Years. Journal of Ecology and Rural Environment, 2015, 31(1):22–29.

[40] Lan-juan LI, Hong REN. Infectious diseases. Beijing: People’s Medical Publishing House, 2013: 172. 10.1186/1471-2334-13-172

[41] Meng-dong LI, Yu-ming WANG. Practice of infectious diseases. Beijing: People’s Medical Publishing House, 2004: 817.

[42] LI X H, CHRISTAKOS G, LIAO Y L, et al. Geographical detectors-based health risk assessment and its application in the neural tube defects study of the Heshun region, China. International Journal of Geographical Information Science, 2010, 24(1): 107–127.

[43] Huang J X, Wang J F, Bo Y C, et al. Identification of health risks of hand, foot and mouth disease in China using the geographical detector technique. International Journal of Environmental Research and Public Health, 2014, 11: 3407–3423. 10.3390/ijerph110303407 24662999 PMC3987041

[44] Guo-liang YU, Jian-hua LI, Jia-xin SUN, et al. Research on BMI influencing factors of urban male adolescents in China based on geographical detector. Geography Research, 2019, 38(8): 2288–2301.

[45] Ren YANG, Yan-sui LIU, Hua-lou LONG, et al. Spatial distribution characteristics and optimized reconstructing analysis of rural settlement in China. Scientia Geographica Sinica, 2016, 36(2): 170–17.

